# Physicochemical and Biological Characterization of the TLR7 Agonist SZU-106

**DOI:** 10.1007/s11095-026-04031-5

**Published:** 2026-02-26

**Authors:** Martin Herbst, Johannes Kipp, Sonja M. Kessler, Christian Wischke

**Affiliations:** Institute of Pharmacy, Martin-Luther-University Halle-Wittenberg, Kurt-Mothes-Str. 3, 06120 Halle, Germany

**Keywords:** cytotoxicity assay, drug stability, Toll-like receptor 7 (TLR7) agonist

## Abstract

**Objective:**

Toll-like receptor 7 (TLR7) recognizes single-stranded RNA and plays a crucial role in initiating immune responses against viral pathogens. This study characterizes SZU-106, a recently developed Toll-like receptor 7 (TLR7) agonist, focusing on its physicochemical and biological properties as key determinants for assessing its suitability for drug product development.

**Methods:**

Distribution coefficients (LogD) of SZU-106 were determined in silico and confirmed experimentally by the shake-flask method. Stress stability tests were performed by incubation at different pH conditions using temperatures between room temperature and 60°C. HPLC ESI–MS/MS analysis was applied to determine the main degradation products. Cytotoxicity tests with different reporter cell lines allowed excluding TRL7-mediated as well as unspecific cytotoxicity in THP-1 monocytes and macrophages.

**Results:**

The analysis of distribution coefficients conducted at pH 5.6 and 7.4 confirmed the predominantly hydrophilic nature of SZU-106. Stress stability testing revealed the stability of SZU-106 in neutral aqueous solutions, while rapid degradation was noted under acidic and basic conditions with rate constants of 2.21·10–3 d-1 to 0.39 h-1. HPLC ESI–MS/MS analysis showed that SZU-106 primarily degrades via hydrolytic cleavage of its amide bonds, with four major degradation products identified and structurally characterized. Cytotoxicity assays with THP-1 monocytes and differentiated macrophages at increasing drug concentrations (10 -1000 µM) illustrated no off-target cytotoxicity and only mild, time-dependent, receptor-mediated effects in TLR7-overexpressing THP-1 cells at high SZU-106 concentrations.

**Conclusions:**

This evaluation of SZU-106 supports its further development as a therapeutic TLR7 agonist, with formulation strategies representing the next stage for drug development.

**Graphical Abstract:**

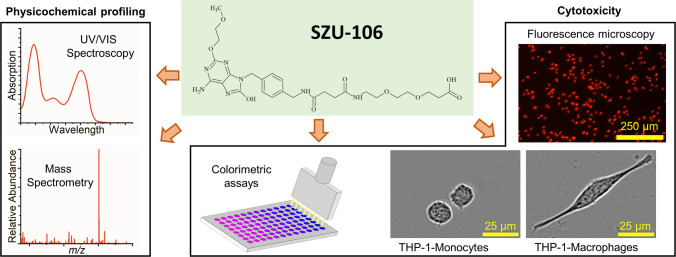

**Supplementary Information:**

The online version contains supplementary material available at 10.1007/s11095-026-04031-5.

## Introduction

Toll-like receptors (TLRs) play a critical role in the innate immune system. As part of the system of pattern recognition receptors (PRRs), TLRs are responsible for recognizing and responding to a variety of pathogens, including those of bacterial and viral origin. They detect pathogens through pathogen-associated molecular patterns (PAMPs) [1, 2], which vary between the different types of TLRs. The TLRs located on the outer cell membrane typically respond to components of foreign cell membranes like peptidoglycans, lipopolysaccharides (LPS), or flagellin. In contrast, the TLRs in the endosomal compartments of immune cells respond to atypical types of invaded nucleic acids, such as single- and double-stranded RNA or unmethylated CpG-DNA [2].

An important subtype of the endosomal TLRs is Toll-like receptor 7 (TLR7). It plays a central role in this system, recognizing single-stranded RNA (ssRNA) and initiating immune responses against viral pathogens. TLR7 is expressed in various types of immune cells, including dendritic cells (DCs), monocytes, macrophages, and B cells [3–5]. These cells are essential for the early detection of viral infections and the initiation of immune responses. Accordingly, TLR7 agonists are of interest as candidates for immunotherapy, oncology, and vaccination.

The potential use of TLR7 agonists in drug products can be justified by their expected effects mediated by a cascade of immune signalling pathways, which culminate in the production and secretion of cytokines, which are essential for coordinating an effective further immune response via cell–cell communication. Specifically, upon activation of the receptor, the adapter protein Myeloid Differentiation Primary Response 88 (MyD88) interacts with members of the IL-1 receptor-associated kinase (IRAK) protein family. This interaction initiates a series of steps that lead to the activation of the transcription factors: nuclear factor 'kappa-light-chain-enhancer' of activated B-cells (NF-κB), Interferon regulatory factor (IRF), and activator protein 1 (AP-1). These transcription factors promote the release of interferon alpha (IFNα) and interferon beta (IFNβ), as well as the production of pro-inflammatory cytokines including tumor necrosis factor alpha (TNFα), pro-interleukin-1β (pro-IL-1β), and pro-IL-18. The latter two require an additional cleavage and activation through caspase 1 to release the mature interleukins [2, 6–8].

In recent years, several TLR7 agonists have been developed and investigated for their ability to modulate immune responses. Among them, imiquimod and resiquimod are the most well-known and have already been approved by regulatory authorities such as the FDA and EMA [6, 9, 10]. These compounds have demonstrated valuable immunostimulatory effects. However, they also present certain limitations. For instance, resiquimod acts as a dual agonist on both TLR7 and TLR8, which may not always be desirable depending on the therapeutic context. Additionally, both imiquimod and resiquimod are relatively lipophilic and poorly soluble in water, which can limit their bioavailability and clinical application [11]. Some newer ligands have demonstrated effective cytokine induction both *in vitro* and *in vivo*, but their administration has been associated with severe systemic side effects [12]. This highlights the need for TLR7 ligands with improved safety profiles and appropriate physicochemical properties.

Based on their chemical structure, TLR7 agonists can be classified into several groups. On the one hand, they are categorized as macromolecular agonists and small-molecule ligands. Macromolecules are typically thymidine or RNA-based structures, which are often not selective for TLR7 [6]. On the other hand, the larger group of small molecules can be further classified according to their aromatic ring systems into (hetero) tri-, bi-, and monocyclic structures [6, 13]. Hetero tricyclic imidazoquinolines, such as imiquimod and resiquimod, were the first synthetically developed TLR7 agonists and remain the only substances approved for clinical use. Imidazoquinolines are mostly dual agonists of TLR7 and TLR8 [13]. A reduction of the ring system from heterotricyclic to hetero bicyclic compounds can produce 6 + 7, 6 + 6, and 6 + 5 fused ring systems that show differences in biological properties. For instance, compounds with 6 + 5 systems typically show higher potency for TLR7 over TLR8, often even without any TLR8 activity, whereas 6 + 6 systems tend to favour TLR8 selectivity [13]. Monocyclic compounds are derivatives of pyrimidine or aminoimidazole, representing the smallest known molecular weight of TLR7 agonists [6, 13]. Especially in the case of the 6 + 5 bicyclic purine derivatives and the pyrimidine-based monocyclic compounds, the structural relationship to natural ssRNA ligands is clearly visible.

**Fig. 1:**
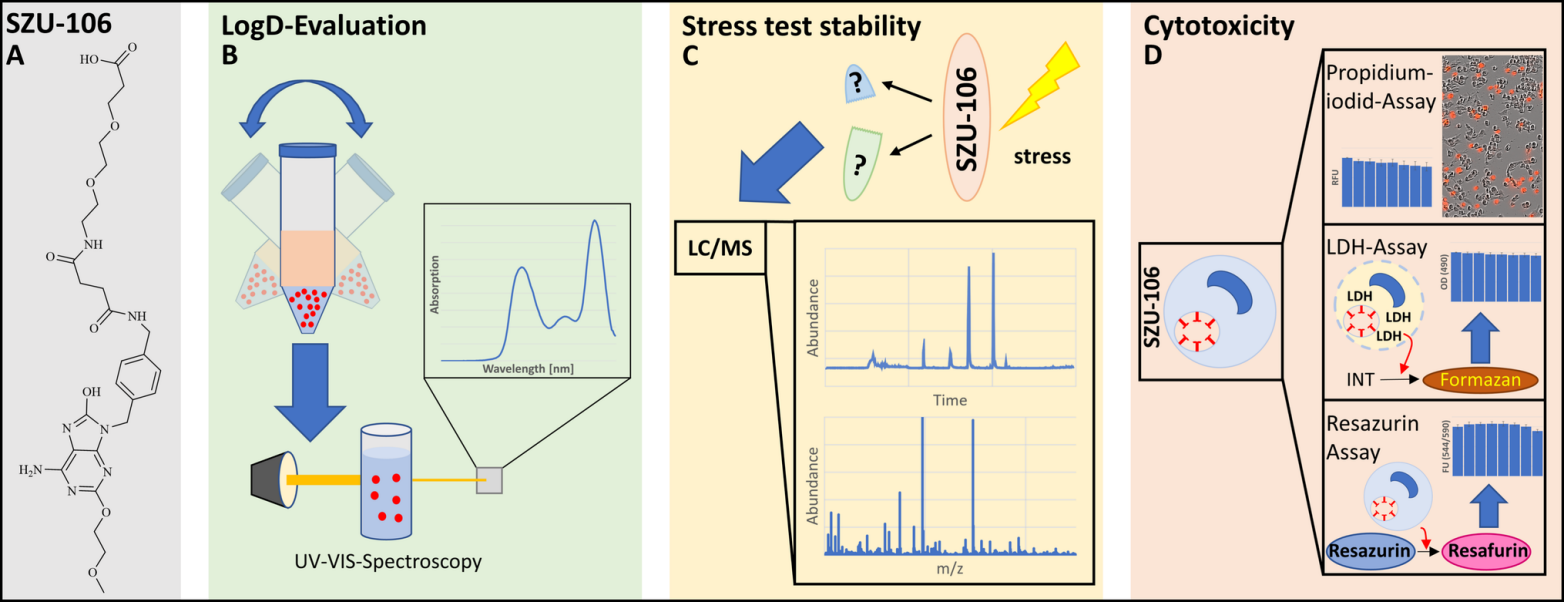
Investigation of physicochemical and biological properties of SZU-106 (**A**: structure of SZU-106) for assessing its suitability for drug product development. The characterization includes (**B**) phase distribution pattern as relevant for membrane diffusion/bioavailability, (**C**) stability studies to understand degradation pathways and kinetics, as well as suitable processing and storage environments, and (**D**) evaluation of unspecific and target-mediated cytotoxicity with different sets of biological readouts.

Within the purine base derivatives, 8-hydroxyadenine analogues have emerged as a particularly promising class [6, 12, 14]. Several compounds from this group have demonstrated immunostimulatory effects comparable to, or even exceeding, those of imiquimod. A recently discovered TLR7 agonist from the group of 8-hydroxyadenine analogues, first reported by Yu and coworkers [15], is SZU-106 (Fig. 1). This compound has been proposed for evaluation as part of an anti-tumor vaccine [15, 16]. However, as a TLR agonist, SZU-106 may be useful in a broader range of applications. For instance, it may also be considered as a candidate adjuvant additive for conventional vaccinations against infectious diseases [4, 5]. Furthermore, TLR7 agonists are of interest for the treatment of allergic diseases, especially bronchial asthma [17, 18]. Additionally, the structure contains an imidazole core. Recent studies on small molecules featuring this motif have highlighted their potential role in antitumor immunology [19].

So far, only limited data are available about the properties of SZU-106; however, a more comprehensive picture will be key to understanding its suitability for making stable, safe, and efficient drug products. In particular, physicochemical data such as the distribution coefficient, as well as stability data of the substance, are not yet reported. Furthermore, there are no available data about the cytotoxicity of the substance. Therefore, this study aims to expand the knowledge on SZU-106 by providing further insights into its properties, including hydrophilicity/lipophilicity, stress stability in different pH regimes, and cytotoxicity (Fig. 1). This evaluation will enhance the understanding of SZU-106, providing essential insights for its further development, including formulation and delivery strategies.

## Materials and Methods

### Materials

SZU-106 was obtained from Enamine Ltd. by custom synthesis (Kyiv, Ukraine). Purified water was prepared by a TKA MicroPure system. Phosphate-buffered saline (PBS) pH 7.4 was prepared using PBS-tablets from Sigma-Aldrich/Merck KGaA (Darmstadt, Germany) and purified water. PBS pH 5.6 and PBS pH 6.4 were prepared following the Ph. Eur. 11.0. Sodium dihydrogen phosphate and disodium hydrogen phosphate were obtained from VWR International LLC (Leuven, Belgium), sodium chloride from CHEMSOLUTE/Th. Geyer GmbH & Co.KG (Renningen, Germany), dipotassium hydrogen phosphate from Sigma-Aldrich/Merck KGaA (Darmstadt, Germany), and potassium dihydrogen phosphate from Grüssing GmbH (Filsum, Germany). *n*-Octanol and dimethyl sulfoxide (DMSO) were purchased from CHEMSOLUTE/Th. Geyer GmbH & Co.KG (Renningen, Germany). Propidium iodide (PI), *L*-(+)-lactic acid, and Tris–HCl were bought from Carl Roth GmbH + Co. KG (Karlsruhe, Germany), poly-*L*-ornithin, resazurin, and Triton-X-100 were from Sigma-Aldrich/Merck KGaA (Darmstadt, Germany), and phorbol 12-myristate-13-acetate (PMA) from EMD Millipore Corp. (Burlington, VT, USA). 2-p-iodophenyl-3-p-nitrophenyl tetrazolium chloride (INT), β-nicotinamide adenine dinucleotide sodium (β-NAD), and 1-methoxyphenazine methosulfate (MPMS) were from MedChemExpress LLC (Monmouth Junction, NJ, USA).

### Evaluation of Distribution Coefficient (LogD)

In order to estimate the LogD in silico, a calculation was performed using the LogD Plug-In for Chemaxon Playground v1.6.1 (ChemAxon Ltd., Budapest, Hungary).

To experimentally evaluate the distribution coefficient of the substance, the shake-flask method was employed. First, a solution of SZU-106 in PBS pH 5.6, 6.4, and 7.4 (each saturated with an appropriate amount of n-octanol) was prepared. 200 µL, 350 µL, or 500 µL of each solution was added to 5 mL *n*-octanol (saturated with the corresponding phosphate buffer) and was vortexed for 30 s. To ensure the equilibration of the system, the mixtures were allowed to rest for 24 h. After this period, samples of the aqueous phase were taken and the amount of SZU-106 in the solution was quantified using UV-spectroscopy (Genesys 50, Thermo Fisher/Life Technologies GmbH; Darmstadt, Germany) and compared to the concentration of the stock solution. The LogD for each pH-value was calculated by the concentrations of SZU-106 in the *n*-octanol and the aqueous phase according to Eq. 1 [20]. Three independent experiments were performed for each pH value.

1 $$logD=\mathrm{log}\left(\frac{{c}_{oct.}}{{c}_{aqu.}}\right)$$

### Stress Stability Testing of SZU-106

SZU-106 was tested under stress conditions to evaluate the degradation mechanism and kinetics of the compound. Aliquots of SZU-106, either dissolved in water, 0.1 M sodium hydroxide (NaOH), or 0.1 M hydrochloric acid (HCl), were incubated at room temperature (21–25°C), at 37 °C, and at 60 °C, respectively.

For the analysis of degradation products, samples were collected after one week of treatment and analyzed by UHPLC-MSMS. The chromatographic separation was performed with an Ultimate 3000 RSLCnano system (Thermo Fisher/Life Technologies Ltd.; Paisley, UK) with a C18 precolumn (Acclaim PepMap 100, 300 μm × 5 mm, 5 μm, 100 Å, Thermo Fisher Scientific) and a self-packed Picofrit (New Objective) nanospray emitter (360 μm OD × 75 μm ID × 150 mm L, 15 μm Tip ID) with C18-stationary phase (3.0 μm, 120 Å, Dr. Maisch GmbH). A flow rate of 300 nl/min with a 20 min linear gradient of water:ACN (10% to 85% B in A) was used, where A is 0.1% (v/v) formic acid and B is 0.1% (v/v) formic acid in ACN. A structural analysis was performed with the coupled LTQ-Orbitrap XL (Thermo Fisher/Life Technologies Ltd.; Paisley, UK), which was operated in the positive mode in an *m/z* range of 150–1,200 at high resolution (120,000 at *m/z* 200). Each second, one MS1 spectra were acquired. The most intense signals were automatically selected for fragmentation by higher-energy collisional dissociation (HCD) at 27% normalized collision energy (NCE).

For compatibility with the analytical system, the samples incubated in NaOH were acidified with HCl before measurement. As controls, untreated aliquots of the same stock of analyte were used, which were stored at −80°C until measurement.

To assess the degradation rate and determine the shelf life, samples were collected at multiple time points up to 35 days (aqueous samples), up to 168 h (acidic solutions), and up to 72 h (basic solutions), respectively. For these quantitative analyses, the Ultimate 3000 RSLC (Thermo Fisher/Life Technologies Ltd.; Paisley, UK) was used with an XBridge C18 column (1 mm X 150 mm, 3.5 µm; Waters, Milford, USA). Separation was performed with a 7 min linear gradient water: ACN (25% to 95% B in A) at a flow rate of 50 µL/min. For mass detection, an LTQ-Orbitrap Fusion spectrometer (Thermo Fisher/Life Technologies Ltd.; Paisley, UK) was employed in the positive mode, *m/z* 180–1200, resolution 60,000 at *m/z* 200.

To quantify the remaining intact SZU-106 ([M + H]^+^ 604.27, [M + 2H]^2+^ 302.64; other ion adducts were negligible), selected ion chromatograms (SIC) were used in the mass ranges 302.6–302.7 m*/z* and 604.2–604.3 m*/z*. The NaOH-treated samples were acidified with HCl before measurement. The remaining amount of intact SZU-106 was normalized to the AUCs determined from the SIC of control samples, which were stored at −80°C. Three independent samples per condition were incubated and used to determine degradation rates. The activation energy and frequency factor of SZU-106 degradation at the different conditions were calculated with the Arrhenius equation.

2 $$k=A\bullet {e}^{\left(-\frac{{E}_{A}}{R \bullet T}\right)}$$

### Cell Cultivation and THP-1 Differentiation

THP-1 cells (#TIB202) were obtained from ATCC, and THP1-Dual-hTLR7 were purchased from Invivogen (Toulouse, France). All cell lines were cultivated in RPMI 1640 + GlutaMAX™ (gibco/Thermo Fisher/Life Technologies Ltd.; Paisley, UK) with 10% fetal bovine serum (Biowest SAS; Nuaillé, France). Cells were kept at 37 °C and 5% CO_2_ in a C170 incubator (Binder GmbH; Tuttlingen, Germany). Transparent, flat-bottom 96-well polystyrene plates (Sarstedt AG & Co. KG; Nümbrecht, Germany) were used for studies with non-differentiated cells after a pre-treatment with poly-L-ornithin 0.01% in order to improve cell adhesion to the plate.

To differentiate THP-1 cells into a macrophage-like state, 40,000 cells per well were seeded into 96-well plates and cultured in medium containing 20 ng/ml PMA for 48 h, then washed twice with PBS and rinsed with fresh medium, and allowed to rest for 24 h before exposure to samples.

### Cytotoxicity Assays

The determination of time-dependent cytotoxicity was performed with a propidium iodide (PI)-based live-cell assay. 40,000 cells/well were seeded in 96-well plates and pretreated with PI (final concentration 2.5 µg/ml), followed by the addition of different concentrations of SZU-106. Control samples either contained 20% DMSO to induce full cell death (FCD; positive control) or cell culture medium to measure untreated cells (negative control). The plates were placed in the IncuCyte S3 (Sartorius AG; Göttingen, Germany) inside an MCO-230 AIC incubator (PHCbi; Tokyo, Japan), and measurements were taken at predefined time points within an incubation period of 24 h. Each measurement per well comprised four pictures in the brightfield and red fluorescence channels at 10 × magnification. The data were analyzed using the IncuCyte 2021 C software. For phase confluence, a representative set of images from each experiment was selected for masking. Objects smaller than 100 µm^2^ were excluded, and segmentation and hole-fill parameters were adjusted as appropriate for each cell type to improve mask accuracy. For red confluence, areas exceeding a threshold of 1 RCU were quantified. To quantify cell death, the ratio between red confluence and total phase confluence was used. The cytotoxicity was calculated according to Eq. 3 using the normalized red fluorescence area *FA* of the different samples.

3 $$Cytotoxicity\; \left(\%\right)=\frac{{FA}_{test}-{FA}_{untreated}}{{FA}_{FCD}-{FA}_{untreated}}\bullet 100 \%$$

For endpoint cytotoxicity assays, 40,000 cells/well were seeded in 96-well plates and treated for 24 h with different concentrations of SZU-106, 20% DMSO (positive control), and cell culture medium (negative control), respectively. Two independent assays, the LDH assay (lactate dehydrogenase) and the resazurin assay, were run in parallel for each sample. For the LDH assay (lactate dehydrogenase), a lysis control (LC; 0% viability, maximum LDH release) was prepared by using one row (6 wells) of untreated cells and exposing them to 1.2% Triton-X-100. Generally, 50 µL supernatant of all samples (including the lysis control) was collected from each well and transferred to another 96-well plate for LDH measurements (details below). The remaining cell suspension was used for the resazurin assay.

5.5 µL resazurin reagent (0.2 mg/ml resazurin salt in PBS pH 7.4) was added per well and incubated for 2 h. Subsequently, the fluorescence was measured with the Cytation 5 (Agilent Technologies, Inc.; Santa Clara, United States). The cytotoxicity was calculated according to Eq. 4 using the fluorescence intensity FI of the different samples.

4 $$Cytotoxicity\; \left(\%\right)=\frac{{FI}_{test}-{FI}_{untreated}}{{FI}_{FCD}-{FI}_{untreated}}\bullet 100 \%$$

For the LDH assay, 50 µL LDH-substrate solution (54 mM lactic acid, 1.3 mM β-NAD^+^, 0.66 mM INT, and 0.014 mM MPMS in 0.2 M Tris–HCl buffer pH 8.2) [21] was added to the cell-free supernatant. After incubation for 15 min, the absorbance was measured with the Cytation 5. The cytotoxicity was calculated according to Eq. 5 using the absorbance A of the different samples.

5 $$Cytotoxicity \;\left(\%\right)=\frac{{A}_{test}-{A}_{untreated}}{{A}_{LC}-{A}_{untreated}}\bullet 100 \%$$

Three independent experiments were performed, and each concentration was tested 6 times per experiment. Mean values and standard deviations were calculated using Microsoft Excel 2021. A Kruskal–Wallis test followed by a Dunn’s post-hoc test was performed with OriginPro 2019.

## Results and Discussion

### Distribution Coefficient

The ability – or inability – of SZU-106 to enter into cells must be considered a crucial factor for its effectiveness in reaching the target TLR7, which is located in the endosomal compartment. In principle, three possible entry routes exist. The first is passive diffusion through the cell membrane (and subsequently through the intracellular membranes). For this pathway, the drug must cross hydrophilic-lipophilic phase boundaries, i.e., from the aqueous extracellular environment into the lipophilic membrane, and then exit the membrane into the hydrophilic intracellular compartment. The second possible way to reach the intracellular space is the uptake via endocytic processes [22]. Two important types of endocytosis are phagocytosis, which mediates the uptake of large particles, and pinocytosis, which encompasses the internalization of fluids and soluble molecules through various pathways [23]. A third possible route is active transport mediated by specific membrane transport proteins.

An established biopharmaceutical parameter, which is used in drug discovery to estimate the ability of new drug compounds to penetrate cell membranes by passive diffusion, is the (pH-dependent) distribution coefficient (LogD) [24]. For the anticipated clinical applications, interesting pH values for analysis of LogD will be the physiological pH of 7.4, but also pH values found in inflamed tissues (pH 6.0 to pH 7.4) or in the tumor microenvironment (pH 6.4 to pH 7.4; as low as pH 5.6 in extreme cases) [25, 26].

LogD values of new compounds can be determined in silico based on the polar surface area of the compound itself, as well as that of the protonated or deprotonated species arising from ionizable groups. This behaviour can be estimated using the molecules’ atomic increments for both the parent compound and its sub-species. The relative abundance of each ionized subspecies is modelled by predicting the pKa values of the compound’s ionizable functional groups. In the case of SZU-106, the LogD values as calculated by Chemaxon software indicated high hydrophilicity, as can be seen from low LogD values of −1.2 at pH 5.6, −2.0 at pH 6.4, and −2.9 at pH 7.4.

**Fig. 2:**
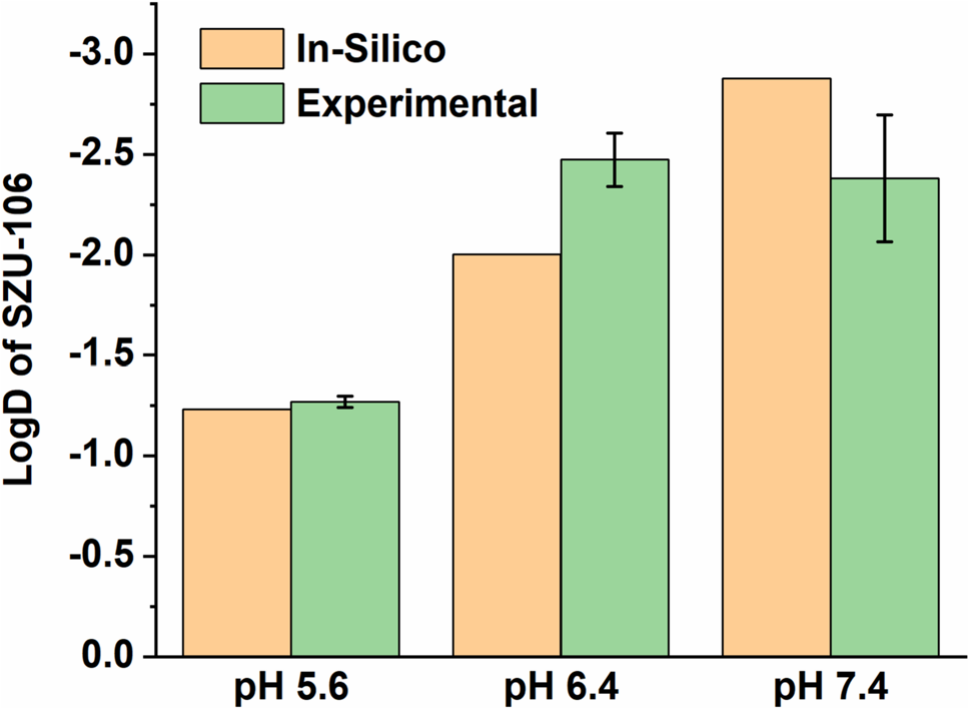
Calculated and experimentally determined LogD values of SZU-106 at the three different pH values 5.6, 6.4, and 7.4. (mean of* n* = 9, error bars represent standard deviation; data were pooled from three independent experiments).

To experimentally confirm the data from in silico predictions, Log D was experimentally determined using the shake-flask method [20, 27, 28], the standard procedure for wet-lab analysis. In cases of hydrophilic drugs with an expected low Log D, it is advisable to employ a two-phase system with a low amount of aqueous phase compared to a relatively high amount of octanol [20]. To ensure a measurable loss of substance from the aqueous to the organic phase, a set of different buffer: octanol ratios was tested, ranging from 1:10 to 1:25. No systematic differences were observed. The experimentally determined LogD values were −1.27 ± 0.03 at pH 5.6, −2.47 ± 0.13 at pH 6.4, and −2.38 ± 0.32 at pH 7.4 (Fig. 2). Despite some deviations at higher pH values, these experimental data strongly confirmed the in silico predictions, highlighting the pronounced hydrophilicity of SZU-106.

The calculated LogD values and the profile of the experimental LogD graph are consistent with expectations based on the molecular structure of the substance with its the carboxyl group (pK_a_ ~ 4.4) (compare Fig. 1). At pH 5.6, the carboxyl group (pK_a_ ~ 4.4) is mostly deprotonated (≈95%), while at pH 6.4, it is nearly fully deprotonated (≈99%), which explains the observed shift towards a more negative LogD. In contrast, the difference in deprotonation of the carboxyl group at pH 6.4 and 7.4 is negligible, which justifies the comparable LogD values at these two pH levels. A possible explanation for the deviation between the experimental and in silico values could be a slight inaccuracy in the calculated pK_a_ of the carboxyl group. A small shift toward a lower pK_a_ would result in a lower LogD, which may explain the higher hydrophilicity observed at pH 6.4 compared with the prediction. This adjustment would also reduce the difference in LogD between pH 6.4 and 7.4, consistent with the experimental data. Furthermore, it should be noted that the simulation does not account for the three-dimensional conformation of the molecule or potential intramolecular hydrogen bonding, both could influence the results [29].

The majority of approved drugs have LogD values between + 0.5 and + 3 [24], highlighting that a certain level of lipophilicity is required for efficient membrane diffusion. If the LogD is too low, the substance will not readily diffuse into the membrane. Conversely, if the LogD is too high, the compound enters the membrane and becomes entrapped within the lipid bilayer, preventing further diffusion into the aqueous intracellular space at relevant amounts [30]. Given the highly hydrophilic nature of SZU-106, it is unlikely to observe relevant levels of penetration into cells by passive diffusion. Accordingly, the realistically possible pathways for cellular uptake are membrane transporters and endocytosis. These pathways are not necessarily dependent on the lipophilicity and seem to be relevant for SZU-106, which has previously shown effects on cells via intracellular TLR7 [15]. So far, there is no information available on transporter proteins for SZU-106. It may be speculated that organic anion transporter proteins (OATPs) could potentially be involved, given their ability to transport anionic molecules with a mass above 350 Da, which, however, typically are amphipathic with a certain hydrophobic region [31]. Overall, endocytic pathways must be considered the most plausible entry route for SZU-106 to reach its intravesicular target, TLR7. In comparison, the already known TLR7 agonist imiquimod or the TLR7/8 agonist resiquimod are clearly more lipophilic, with LogD 2.65 for imiquimod and 1.72 for resiquimod (at pH 5.6, 6.4, and 7.4; LogD values were calculated with Chemaxon LogD Plug-in). Therefore, compared to SZU-106, imiquimod and resiquimod are expected to cross cell membranes by passive diffusion, facilitating absorption and systemic distribution, while alternative pathways and strategies must be considered for SZU-106 during drug development.

The most important target cells for therapeutic concepts involving SZU-106 are monocytes, macrophages, and dendritic cells, which express TLR7 at higher quantities [5]. Importantly, these cells are capable of phagocytosis in addition to pinocytosis. So, a possible strategy to increase the amount of SZU-106 in these cells could be its formulation in particulate carriers, which are phagocytosed by the cells.

### Stress Stability

#### Determination of the Degradation Mechanism

**Fig. 3:**
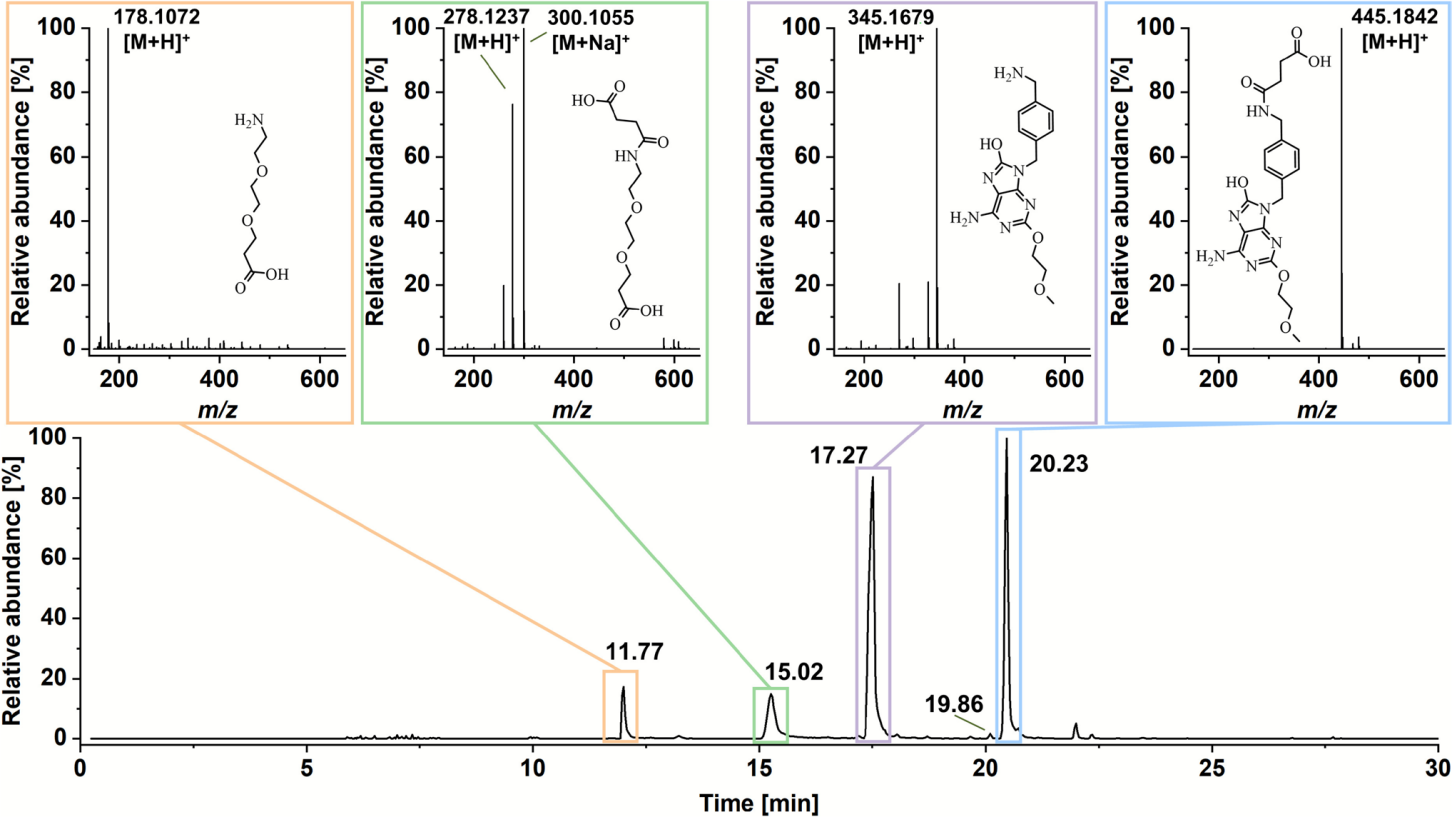
LC–MS total ion chromatogram (TIC) and MS-spectra of the main chromatographic peaks, including assignment of the corresponding compounds. Representative data set for a sample stored in 0.1 M NaOH at 60 °C for 7 days.

For the development of SZU-106 – particularly concerning processing and storage – an understanding of its stability is essential. To explore the stability of SZU-106, stress tests were conducted. Based on the compound’s structure, the most probable degradation mechanism is hydrolysis of the two amide bonds, which is expected to result in four potential degradation products (see structures in Fig. 3). To test this hypothesis, SZU-106 was subjected to various stress conditions, including different temperatures and pH values. Specifically, room temperature (21–25°C) and 60 °C were selected as storage and stress conditions, respectively, while 37 °C was included as a physiologically relevant temperature. The investigation was performed in three different media, water, 0.1 M HCl, and 0.1 M NaOH, as recommended [32].

All four predicted degradation products were detected in the samples (Fig. 3), regardless of the conditions (water, acid, base) used in the stress tests. The primary difference observed under the different conditions was the extent of degradation. These findings prove the hypothesis of hydrolytic cleavage. Using tandem MS, the molecular structures corresponding to the ions observed in MS1 (Fig. 3) could be confirmed by fragmentation (Figs. S1-S6). Interestingly, two of the degradation products (Fig. 3) are already known in the context of TLR7 modulation. The product with a molecular mass of 444.18 Da ([M + H]^+^ 445.1842), known as SZU-101, is a TLR7 ligand and served as the guiding structure for the development of SZU-106 [16, 33]. The second degradation product, with a molecular mass of 344.16 Da ([M + H]^+^ 345.1679), is referred to as SZU-008 T/SZU-142 [34]. This compound is an intermediate in the synthesis of SZU-106 and related substances. While no direct effects on TLR7 have been reported, its 8-hydroxyadenine structure suggests a potential interaction with the receptor. This raises the interesting question of the extent to which a chemical degradation of SZU-106 results in a loss of biological function, which may be clarified in future studies.

#### Determination of Shelf Life

In addition to the qualitative aspects of the degradation mechanism, the quantitative evaluation of SZU-106 degradation is also of particular interest, especially with respect to its shelf life in solution. To this end, the remaining amount of the intact SZU-106 was quantified under several storage conditions. A calibration curve with 16 concentrations ranging from 20 µg/ml to 937.5 pg/ml was used for quantification. Linearity was confirmed by plotting the detector response against the concentration, yielding a correlation coefficient (r^2^) of 0.9991. The Limit of Detection (LOD) and Limit of Quantification (LOQ) were determined according to ICH Guideline Q2 (R2) using the standard deviation of the y-intercept [35]. The LOD was 866.6 pg/ml, and the LOQ was 2.63 ng/ml. These results confirm the method's suitability for determining SZU-106 in the samples, which was sufficiently sensitive to analyze the compound concentrations in the incubated samples ranging from 19.03 µg/ml to 99.75 ng/ml.

**Fig. 4:**
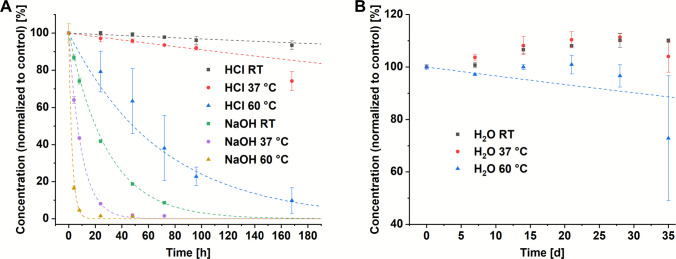
Measured amount of SZU-106 after storage in (**A**) 0.1 M HCl or 0.1 M NaOH and (**B**) H_2_O at room temperature (RT), 37 °C, and 60°C. Dotted lines represent a fit based on the equation: $y=100\bullet {e}^{-k\bullet x}$. (mean of *n* = 3, error bars represent standard deviation, outliers (Dixon test) were removed).

The rate constant *k* for each temperature was determined by regression analysis of the data, assuming first-order kinetics (Fig. 4). Based on these results, the shelf life of SZU-106 for the given storage conditions was calculated (Table 1). In accordance with ICH stability guidelines, a cumulative degradation level of approximately 5% is commonly used as a threshold, corresponding to at least 95% of the drug substance remaining intact, although the exact acceptance criteria are product-specific [36]. Based on this rule, shelf lives of only a few minutes (e.g., SZU-106 in NaOH at 60 °C) up to several days could be calculated. Generally, SZU-106 was much more stable under acidic conditions compared to basic conditions, which is favourable considering the slightly acidic tissue environment in inflammation and cancer tissue. Importantly, for solutions of SZU-106 in pure water, no degradation was observed at room temperature as well as at 37 °C after 35 days of incubation. Therefore, no rate constant could be calculated for these conditions and a minimum shelf life of 35 days is proposed for both.

**Table 1:** Rate constant of degradation and shelf life (> 95% intact drug remaining) of SZU-106 in different solvents at room temperature (RT), 37 °C, and 60 °C (n.a.: not applicable)

	Rate constant	Shelf life
H_2_O, RT	n.a	> 35 d
H_2_O, 37°C	n.a	> 35 d
H_2_O, 60°C	2.2·10^–3^ ± 9.1·10^–4^ d^−1^	23.2 ± 9.6 d
0.1 M HCl, RT	3.2·10^–4^ ± 2.6·10^–5^ h^−1^	160.9 ± 12.9 h
0.1 M HCl, 37°C	9.4·10^–4^ ± 5.7·10^–4^ h^−1^	54.6 ± 3.3 h
0.1 M HCl, 60°C	1.4·10^–2^ ± 9.1·10^–4^ h^−1^	3.6 ± 0.2 h
0.1 M NaOH, RT	3.4·10^–2^ ± 1.9·10^–4^ h^−1^	1.5 ± 0.0 h
0.1 M NaOH, 37°C	1.1·10^–1^ ± 5.5·10^–4^ h^−1^	0.5 ± 0.0 h
0.1 M NaOH, 60°C	3.9·10^–1^ ± 8.9·10^–3^ h^−1^	0.1 ± 0.0 h

The degradation curves well corresponded to a first order kinetics, as can be seen from the curve fitting in Fig. 4. The rate constant k, as determined from these fits for the different temperatures at acidic and basic conditions, allowed to determine the activation energy (E_A_) and frequency factor (A) of the degradation reaction according to Eq. 2. Based on the degradation rates at 37 °C and 60 °C, an E_A_ of approximately 100 kJ·mol^−1^ and an A of around 1.2 · 10^14^ h^−1^ was estimated for SZU-106 in 0.1 M HCl. For 0.1 M NaOH, the calculated E_A_ was ~ 50 kJ·mol^−1^ and the A was ~ 2 · 10^7^ h^−1^. These values fall within the expected range for this type of reaction [37–39].

Overall, these data provide valuable insights into the stability of SZU-106, particularly highlighting its satisfactory shelf life in pure water. Additionally, its stability in even strongly acidic solutions appears sufficient for a wide range of processing steps. However, the lack of stability under basic conditions must be considered, and exposure to basic environments should be minimized. A processing in the presence of strong bases should be entirely avoided due to the significant loss of substance within just a few hours.

### Cytotoxicity

To evaluate the influence of the substance SZU-106 on cell viability and to define a potential critical dose range, a number of different assays were applied along with* in vitro* cell culture tests. As SZU-106 would have its largest relevance in the interaction with immune cells such as monocytes, macrophages, and dendritic cells, the human monocytic cell line THP-1 was selected as an *in vitro* model. THP-1 cells are widely used for mechanistically studying inflammatory processes, for drug testing, and for general exploration of immune responses [40]. THP-1 cells can be differentiated into a macrophage-like state using PMA via well-established protocols [41, 42], which were included in our investigation of compound-induced cytotoxicity.

The cytotoxicity of a substance can be based on two different phenomena: non-specific off-target effects and cytotoxicity attributable to the mechanism of action of the substance. In case of a TLR7 agonist, it is possible that a strong activation of the receptor and the induced inflammatory cascade reaction can lead to cell death, especially at high concentrations of the compound. According to literature, THP-1 cells do not express TLR7 in a relevant manner (Human Protein Atlas) [43] and were therefore used to test for nonspecific cytotoxicity. In addition, we used the cell line THP1-Dual-hTLR7, which stably expresses TLR7 and a mutated variant of the chaperone protein UNC93B1, resulting in an ability for TLR7-specific response.

To measure the concentration-dependent rates of cell death, three different types of assays were used. The resazurin assay detects the amount of active redox systems, which is an indicator of cell viability or, if absent, of cytotoxicity. The LDH test determines the amount of lactate dehydrogenase in the supernatant of the cells, which can be found outside the cells only in case of lytic cell death [44]. In contrast, the PI assay analyzes the red fluorescence of the PI dye that has reached the nucleus and bound to DNA. In living cells, PI is not able to penetrate the cell membrane and the nucleus; therefore, the PI assay is indicative of late apoptotic cells and cells that underwent lytic cell death, in which a red fluorescence signal will be observable [45].

**Fig. 5:**
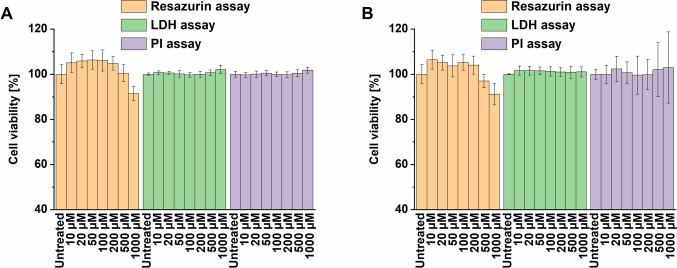
Concentration-dependent unspecific cytotoxicity of SZU-106 on THP-1 cells for (**A**) undifferentiated (monocyte-type) THP-1 cells and (**B**) PMA-differentiated (macrophage-type) THP-1 cells, measured as endpoint viability after 24 h of exposure. The cells were analyzed with the Resazurin, LDH, and PI assays (mean of *n* = 18, error bars represent standard deviation; data from three independent experiments; the data were compared by the Kruskal–Wallis test to the untreated control samples; no significance was found).

The evaluation of nonspecific cytotoxic effects of SZU-106 in THP-1 monocytes revealed only a negligible reduction of cell viability after 24 h of treatment compared to untreated cells, which was only detectable at relatively high concentrations of the analyte. More specifically, in undifferentiated (monocyte-type) THP-1 cells, the cell viability was reduced to 91.4 ± 3.1% at the highest tested concentration of 1000 µM, while no measurable effect was found at lower concentrations of SZU-106. Comparable observations were made for PMA-differentiated (macrophage-type) THP-1, where the cell viability was 91.2 ± 4.7% at 1000 µM and 97 ± 3% at 500 µm. For smaller amounts of SZU-106, again, no trend to lower values in viability was noted. Furthermore, the LDH assay and the PI assay showed no cytotoxic effects (Table S7; Fig. 5). Additionally, no time-dependent effect was observable in the PI assay (Fig. S7). The combination of these results leads to the conclusion that the investigated concentrations of SZU-106 do not induce unspecific cell death. The reduced viability in the resazurin assay could be explained as early-stage cytotoxicity at high substance concentrations. However, a stress-dependent reduction of metabolic activity in the cells would explain the results too. Overall, these findings suggest that SZU-106 does not exhibit off-target cytotoxicity.

**Fig. 6:**
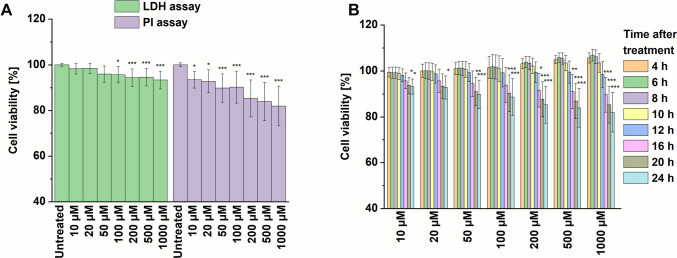
Concentration-dependent specific cytotoxicity of SZU-106 on undifferentiated (monocyte-type) THP1-Dual-hTLR7 cells. (**A**) Endpoint measurement of cell viability after 24 h of incubation as determined with the LDH and the PI assays. (**B**) Concentration-dependency of cytotoxicity measured with PI-staining over several time points (mean of* n* = 18, error bars represent standard deviation, data from three independent; the data were compared by the Kruskal–Wallis test to the untreated control samples; *p < 0.05; **p < 0.01; ***p < 0.001).

In the next step, the TLR7-overexpressing THP1-Dual-hTLR7 cell line was used to measure toxicity associated with TLR activation. It should be noted that cell viability could not be accurately assessed using the resazurin assay in these cells, as, along with a morphological shift towards a more macrophage-like appearance (Fig. S8), the cells showed increased metabolic activity, which falsified the resazurin assay results (Fig. S9). However, the LDH and PI assays were suitable for evaluating the specific cytotoxicity of SZU-106 in the TLR7-expressing THP1 cells. In general, the cells displayed a more evident dose-dependent cytotoxic response to SZU-106 (Fig. 6). In the case of the PI assay, cell viability was affected even at the lowest substance concentration of 10 µM (93.5% ± 3.6%), and increased with rising concentrations of SZU-106 (Table S8; Fig. 6A). In all tested concentrations, viability remained > 80%, which is acceptable. In the LDH assay, we observed a similar trend, although to an even lesser extent, with > 93% viable cells at the highest SZU-107 concentration. This pattern indicates that only a few cells exhibited cell lysis. In combination with the results from the THP-1 cells, these findings strongly suggest that the mild cytotoxicity is TLR7-dependent and not due to any nonspecific toxicity of the compound.

Besides the observations at the endpoint of the incubation period, the time dependency of the cytotoxic effect was also investigated for THP1-Dual-hTLR7 using the PI assay. In the case of TLR7 overexpressing cells, the accumulation of dead cells started after around 8 h. Interestingly, this onset of cytotoxicity was independent of the used concentration (Fig. 6B).

The observed delayed accumulation of PI-positive cells suggests a progressive loss of cell membrane integrity over time, arguing against immediate necrosis, which typically results in rapid lysis. Instead, this temporal pattern aligns with either apoptosis followed by secondary necrosis or necroptosis, a form of regulated necrosis-like cell death [46, 47]. Notably, necroptosis appears to be a particularly plausible mechanism in response to TLR7 activation, as it ultimately induces the secretion of TNFα, a key trigger of necroptotic cell death [48]. Furthermore, the delayed cytotoxicity aligns with the temporal requirements of TLR7-mediated signaling, including NF-κB activation, transcription, and translation of pro-inflammatory cytokines, and subsequent induction of cell death pathways.

According to these results, a possible dosing range of the substance appears to be limited only by the receptor-mediated effects in the cells, which is an important finding. Given the moderate cell toxicity, the maximum dose could even reach the highest tested concentration of 1000 µM, with more than 80% of cells being viable. These findings should also be set in the context of previous findings, which showed relevant activation of the receptor and subsequent cytokine secretion for concentrations of 20 µM and below [16], as well as our own experiments showing substantial cellular activation at concentrations below 10 µM in THP1-Dual-hTLR7 cells (Fig. S10). Thus, concentrations inducing the desired functional responses in target cells are much lower than the concentration range that is required to induce a (only) moderate toxicity, which is advantageous and may be relevant in terms of compound safety during its subsequent development.

## Conclusion

The physicochemical and biological evaluation of SZU-106 as a novel TLR7 agonist highlights key properties of this compound, which are essential for guiding its further development. Its hydrophilic nature, as evidenced by the here-reported LogD, suggests limited passive diffusion across cellular membranes, indicating that the compound would benefit from formulation strategies that enhance the delivery to its intracellular target.

Additionally, stress tests for aqueous stability were promising, while strongly basic conditions were found to compromise stability and should be avoided. Interestingly, some of the degradation products – whose structures were proposed and confirmed here – are themselves TLR7 ligands and thus retain bioactivity.

Cytotoxicity studies identified a moderate cytotoxic effect attributed to TLR7 activation only at high concentrations, while no off-target cytotoxicity was detected* in vitro* in THP-1 cells.

These findings provide a solid foundation for further studies to develop an effective formulation or delivery system for SZU-106, to evaluate its immune-stimulatory efficacy and its therapeutic potential in greater depth in the future.

## Supplementary Information

Below is the link to the electronic supplementary material. 

ESM 1 (PDF 1.20 MB) 

## Data Availability

The authors declare that the data supporting the findings of this study are available within the paper and its Supplementary Information files. Should any data be needed in another format they are available from the corresponding author upon reasonable request.
