# Supplementary material for: Physicochemical and Biological Characterization of the TLR7 Agonist SZU-106
Source: Pharm Res. 2026 Feb 26;43(4):1177–88. doi: 10.1007/s11095-026-04031-5 (PMC13179205; doi:10.1007/s11095-026-04031-5)
Supplement: Supplementary file 1 — (PDF 1.20 MB) [file 11095_2026_4031_MOESM1_ESM.pdf]

Supplementary data

Physicochemical and Biological Characterization of the TLR7 Agonist SZU-106

Martin Herbst [a], Johannes Kipp [a], Sonja Kessler [a], Christian Wischke [a,*]

[a] *Institute of Pharmacy, Martin-Luther-University Halle-Wittenberg, Kurt-Mothes-Str. 3, Halle 06120, Germany*

* Corresponding author: Christian Wischke
E-mail address: Christian.wischke@pharmazie.uni-halle.de

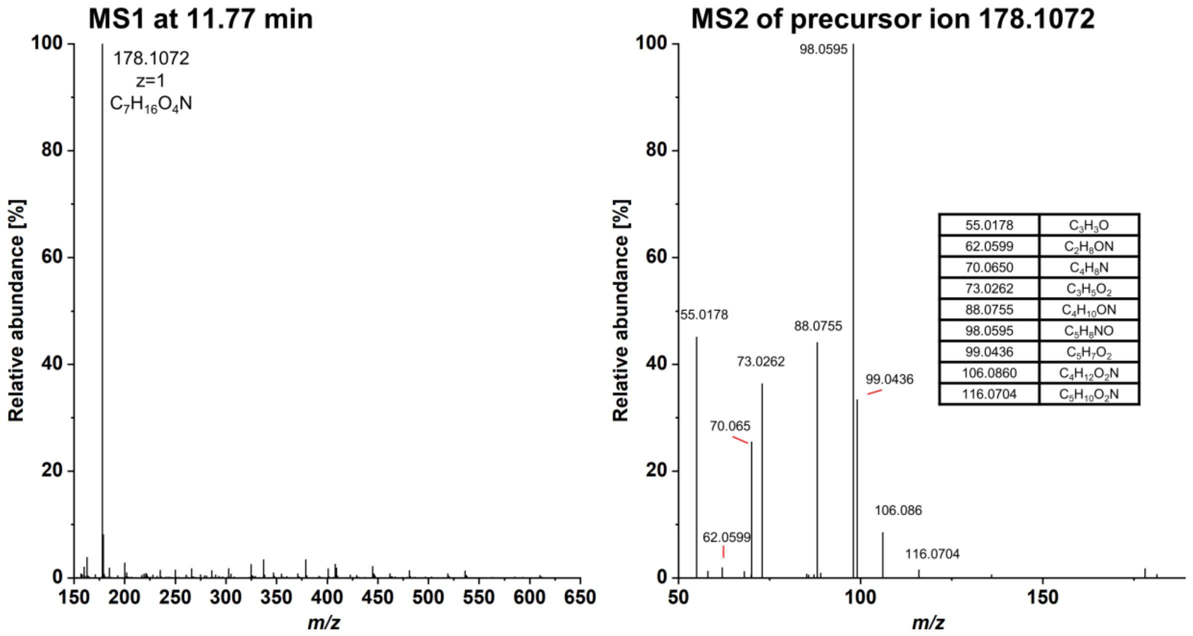

Fig. S1: MS spectra (left) of the chromatographic peak at a retention time of 11.77 min and MS/MS spectra (right) for the fragmentation of the mother ion m/z 178.1072. Representative data obtained from a sample incubated in 0.1 M NaOH at 60°C for 7 days.

Table S1: Proposed sum formula and structural assignments of the mother ion and MS/MS fragments based on the signals presented in Fig. S1.

178.1072	$C_7H_{16}O_4N$	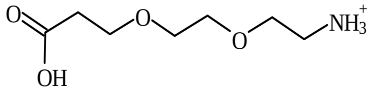
55.0178	C_3H_3O	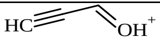
62.0599	C_2H_8ON	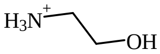
70.065	C_4H_8N *	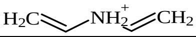
73.0262	$C_3H_5O_2$	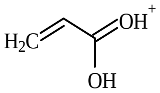
88.0755	$C_4H_{10}ON$	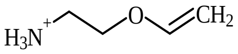
98.0595	C_5H_8NO *	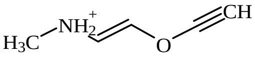
99.0436	$C_5H_7O_2$	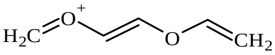
106.086	$C_4H_{12}O_2N$	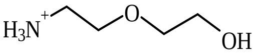
116.0704	$C_5H_{10}O_2N$	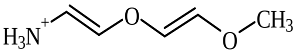

* Hypothetical structure based on fragment mass. Complex rearrangements during fragmentation might produce these structures

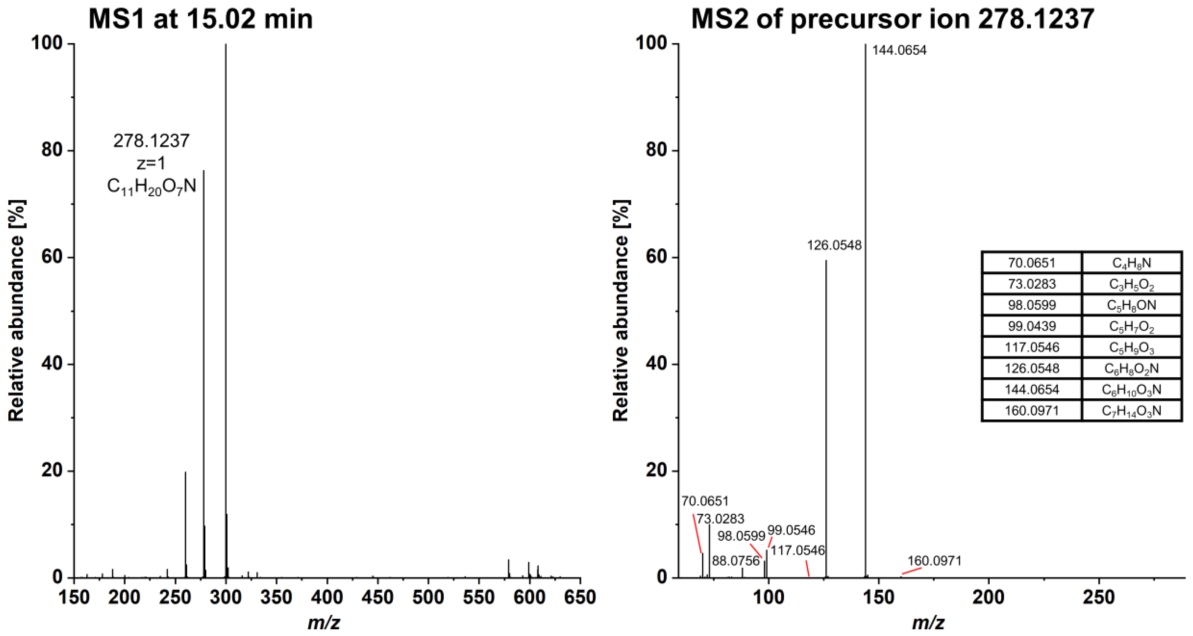

Fig. S2: MS spectra (left) of the chromatographic peak at a retention time of 15.02 min and MS/MS spectra (right) for the fragmentation of the mother ion m/z 278.1237. Representative data obtained from a sample incubated in 0.1 M NaOH at 60°C for 7 days.

Table S2: Proposed sum formula and structural assignments of the mother ion and MS/MS fragments based on the signals presented in Fig. S2.

278.1237	$C_{11}H_{20}O_7N$	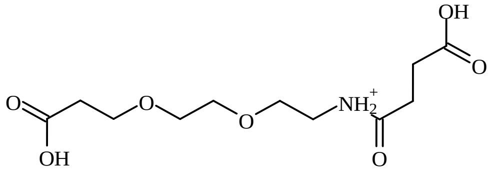
70.0651	C_4H_8N	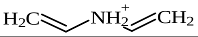
73.0283	$C_3H_5O_2$	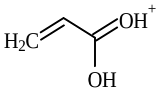
98.0599	C_5H_8ON	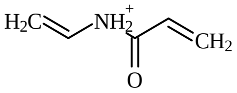
99.0439	$C_5H_7O_2$	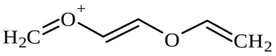
117.0546	$C_5H_9O_3$	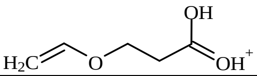
126.0548	$C_6H_8O_2N$	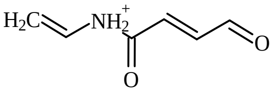
144.0654	$C_6H_{10}O_3N$	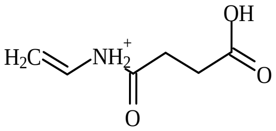
160.0971	$C_7H_{14}O_3N$	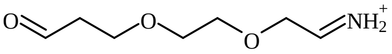

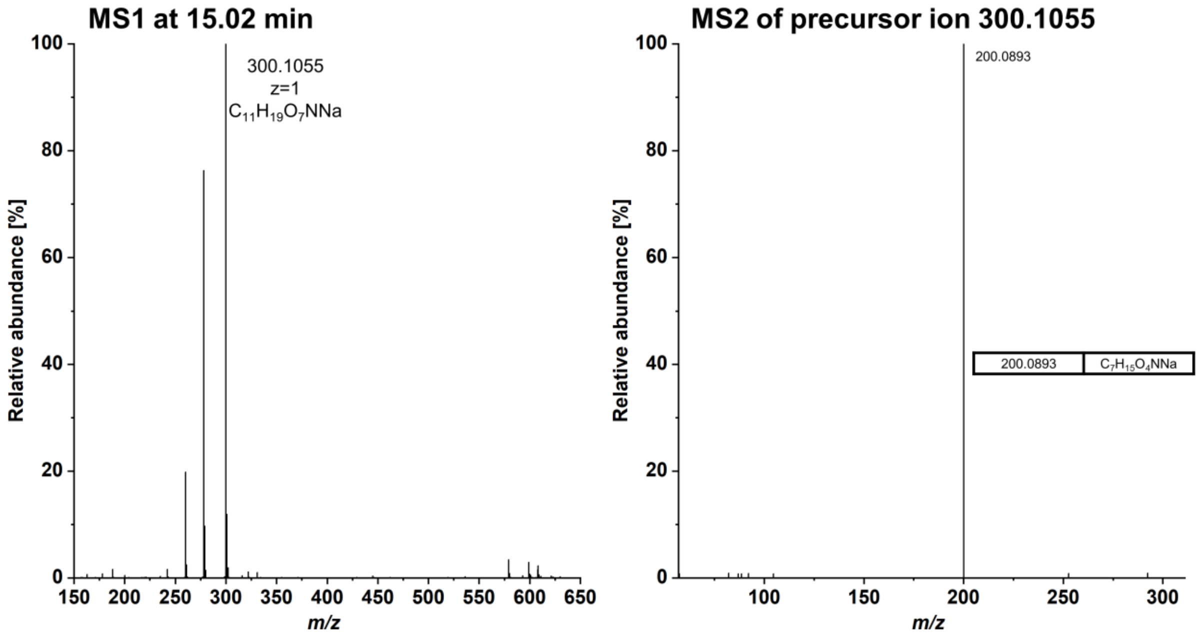

Fig. S3: MS spectra (left) of the chromatographic peak at a retention time of 15.02 min and MS/MS spectra (right) obtained upon fragmentation of the mother ion m/z 300.1055. Representative data obtained from a sample incubated in 0.1 M NaOH at 60°C for 7 days.

Table S3: Proposed sum formula and structural assignments of the mother ion and MS/MS fragments based on the signals presented in Fig. S3.

300.1055	$C_{11}H_{19}O_7NNa$	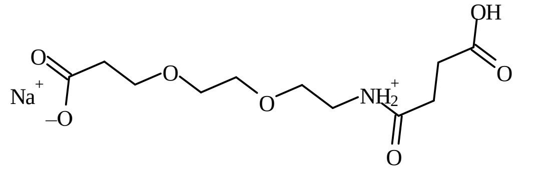
200.0893	$C_7H_{15}O_4NNa$	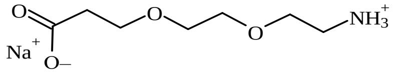

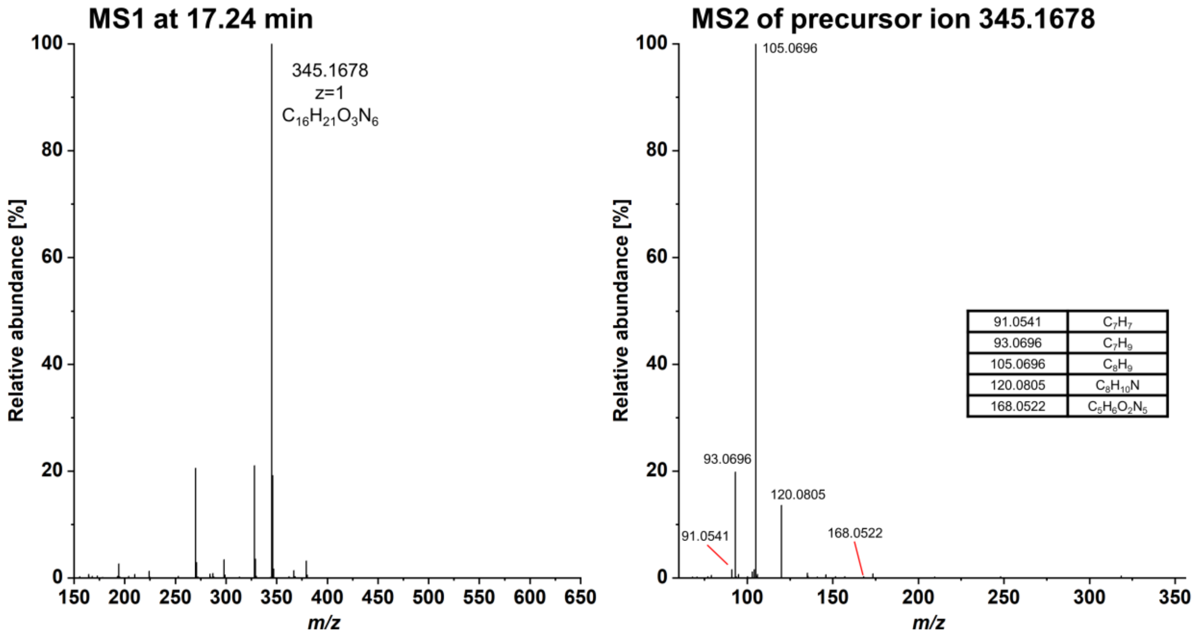

Fig. S4: MS spectra (left) of the chromatographic peak at a retention time of 17.24 min and MS/MS spectra (right) obtained upon fragmentation of the mother ion m/z 345.1678. Representative data obtained from a sample incubated in 0.1 M NaOH at 60°C for 7 days.

Table S4: Proposed sum formula and structural assignments of the mother ion and MS/MS fragments based on the signals presented in Fig. S4.

345.1678	$C_{16}H_{21}O_3N_6$	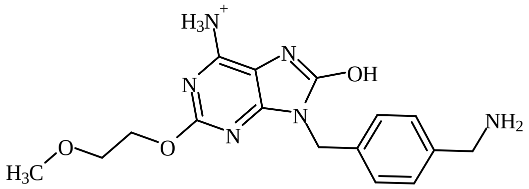
91.0541	C_7H_7	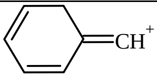
93.0696	C_7H_9	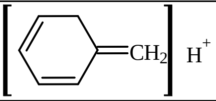
105.0696	C_8H_9	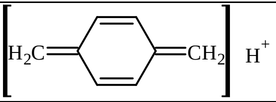
120.0805	$C_8H_{10}N$	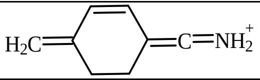
168.0522	$C_5H_6O_2N_5$	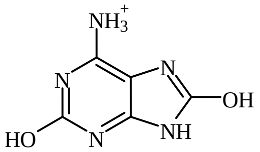

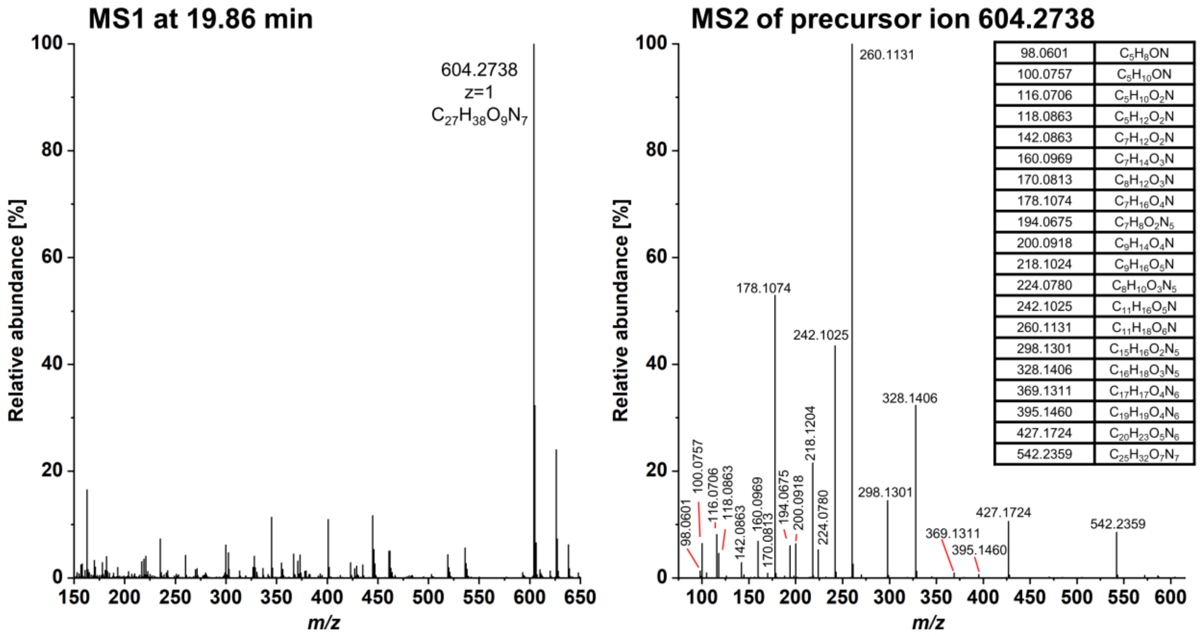

Fig. S5: MS spectra (left) of the chromatographic peak at a retention time of 19.86 min and MS/MS spectra (right) obtained upon fragmentation of the mother ion m/z 604.2738. Representative data obtained from a sample incubated in 0.1 M NaOH at 60°C for 7 days.

Table S5: Proposed sum formula and structural assignments of the mother ion and MS/MS fragments based on the signals presented in Fig. S5.

604.2738	$C_{27}H_{38}O_9N_7$	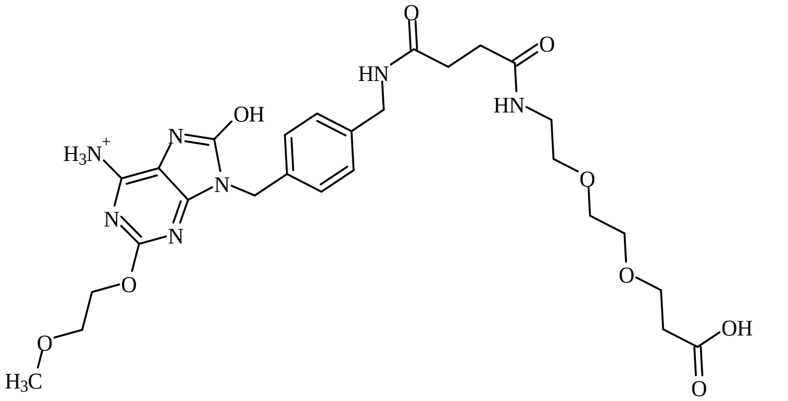
98.0601	C_5H_8ON	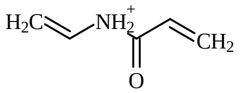
100.0757	$C_5H_{10}ON$	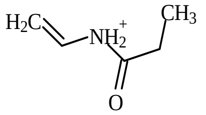
116.0706	$C_5H_{10}O_2N$	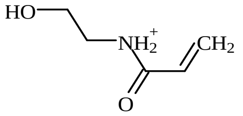
118.0863	$C_5H_{12}O_2N$	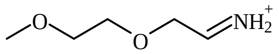
142.0863	$C_7H_{12}O_2N$	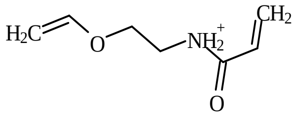

160.0969	$C_7H_{14}O_3N$	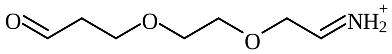
170.0813	$C_8H_{12}O_3N$	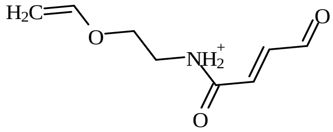
178.1074	$C_7H_{16}O_4N$	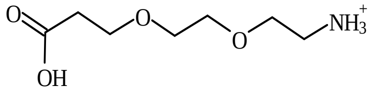
194.0675	$C_7H_8O_2N_5$	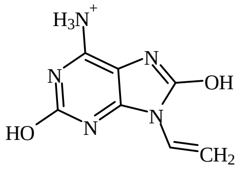
200.0918	$C_9H_{14}O_4N$	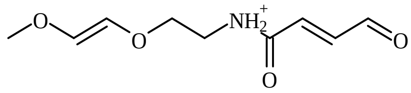
218.1024	$C_9H_{16}O_5N$	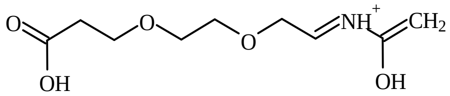
224.078	$C_8H_{10}O_3N_5$	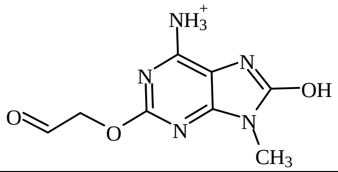
242.1025	$C_{11}H_{16}O_5N$	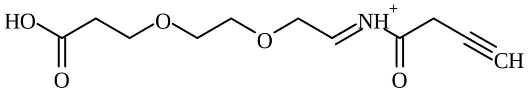
260.1131	$C_{11}H_{18}O_6N$	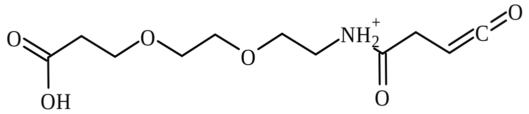
298.1301	$C_{15}H_{16}O_2N_5$	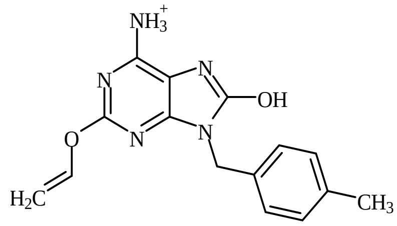
328.1406	$C_{16}H_{18}O_3N_5$	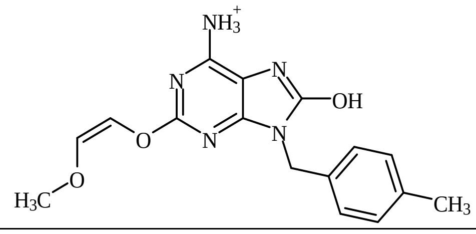
369.1311	$C_{17}H_{17}O_4N_6$	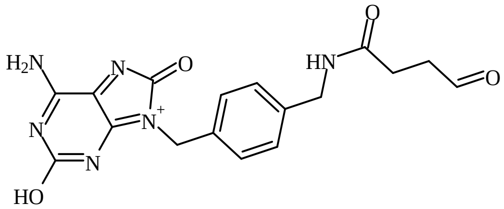

395.146	$C_{19}H_{19}O_4N_6$	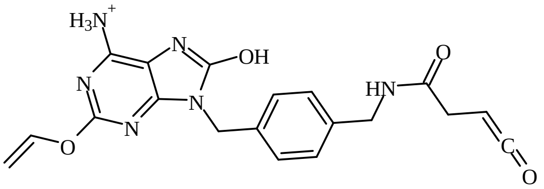
427.1724	$C_{20}H_{23}O_5N_6$	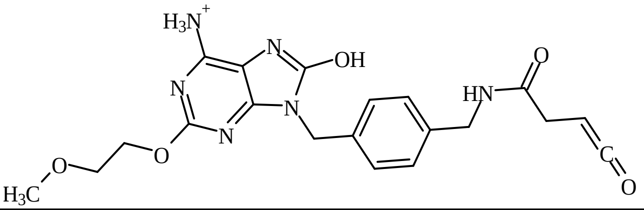
542.2359	$C_{25}H_{32}O_7N_7$	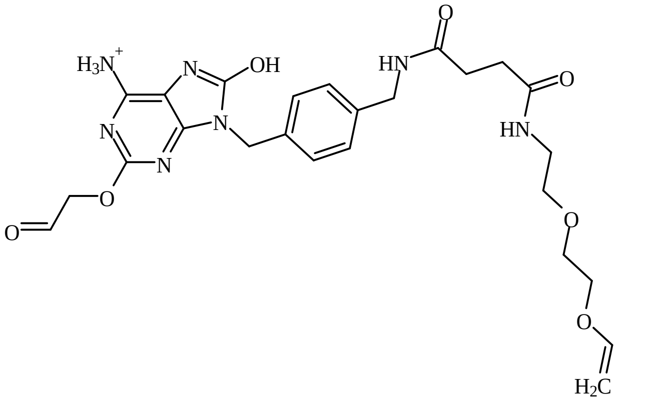

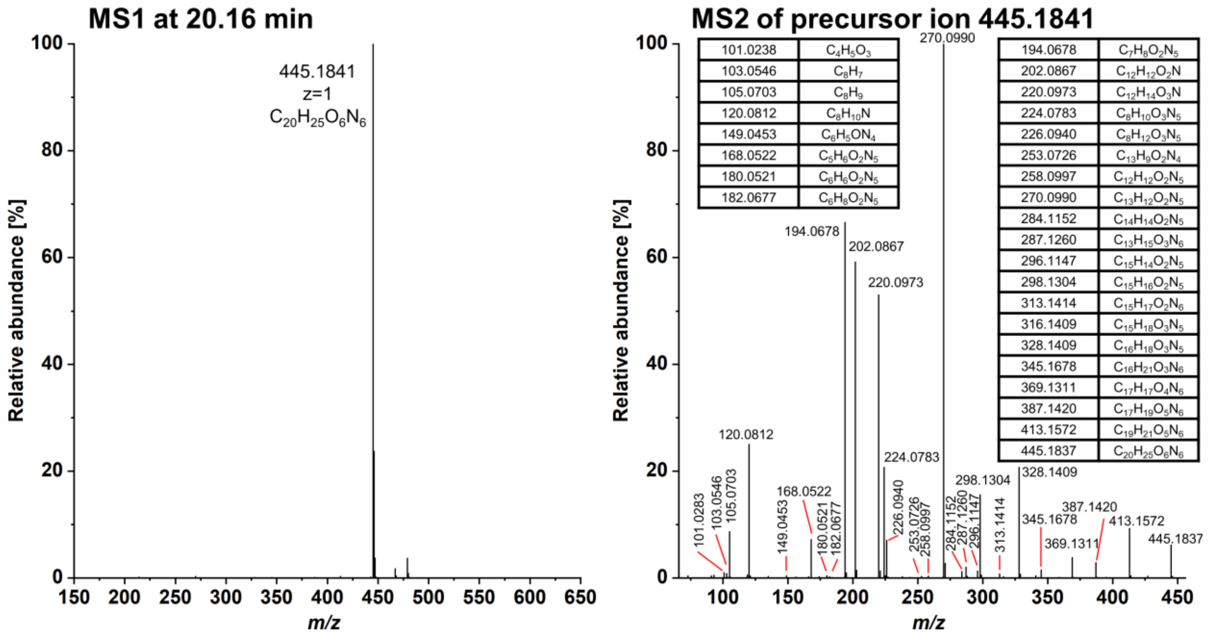

Fig. S6: MS spectra (left) of the chromatographic peak at a retention time of 20.16 min and MS/MS spectra (right) obtained upon fragmentation of the mother ion m/z 445.1841. Representative data obtained from a sample incubated in 0.1 M NaOH at 60°C for 7 days.

Table S6: Proposed sum formula and structural assignments of the mother ion and MS/MS fragments based on the signals presented in Fig. S6.

445.1841	$C_{20}H_{24}O_6N_6$	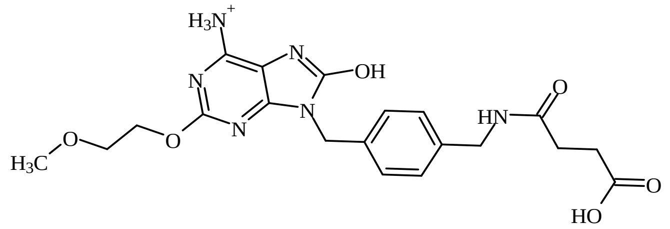
101.0238	$C_4H_5O_3$	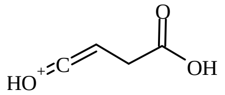
103.0546	C_8H_7	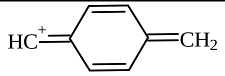
105.0703	C_8H_9	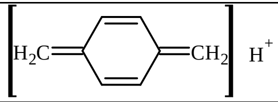
120.0812	$C_8H_{10}N$	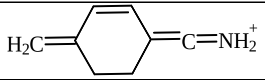
149.0453	$C_6H_5ON_4$	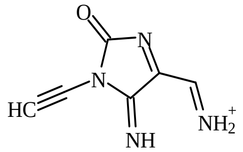
168.0522	$C_5H_6O_2N_5$	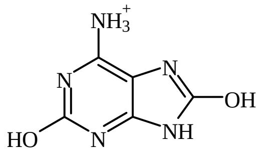

180.0521	$C_6H_6O_2N_5$	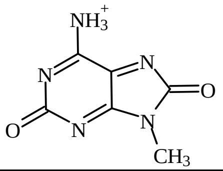
182.0677	$C_6H_8O_2N_5$	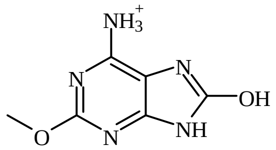
194.0678	$C_7H_8O_2N_5$	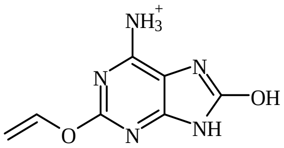
202.0867	$C_{12}H_{12}O_2N$	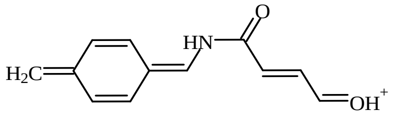
220.0973	$C_{12}H_{14}O_3N$	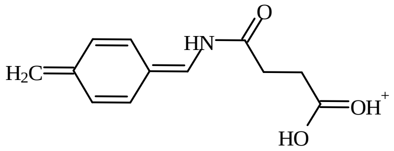
224.0783	$C_8H_{10}O_3N_5$	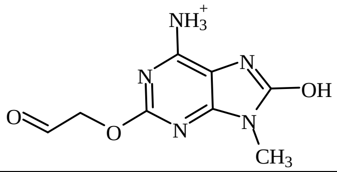
226.094	$C_8H_{12}O_3N_5$	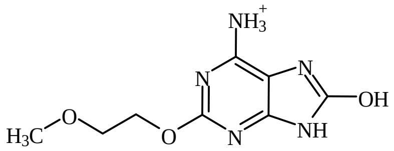
253.0726	$C_{13}H_9O_2N_4$	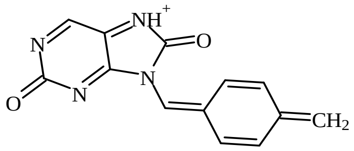
258.0997	$C_{12}H_{12}O_2N_5$	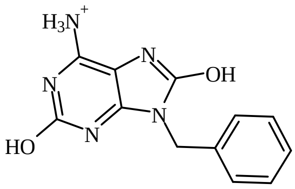
270.099	$C_{13}H_{12}O_2N_5$	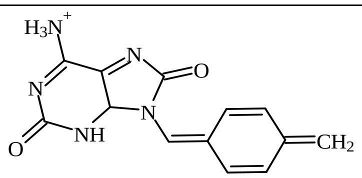
284.1152	$C_{14}H_{14}O_2N_5$	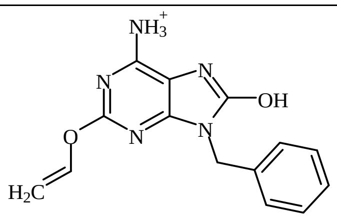

287.126	$C_{13}H_{15}O_2N_6$	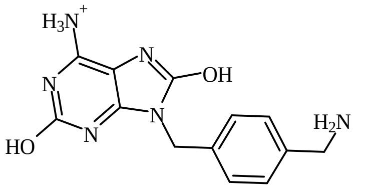
296.1147	$C_{15}H_{14}O_2N_5$	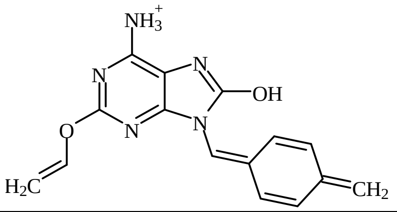
298.1304	$C_{15}H_{16}O_2N_5$	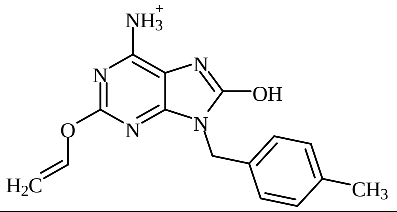
313.1414	$C_{15}H_{17}O_2N_6$	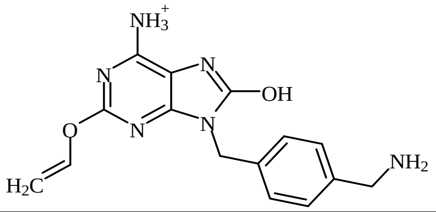
316.1409	$C_{15}H_{18}O_3N_5$	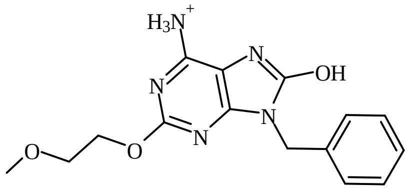
328.1409	$C_{16}H_{18}O_3N_5$	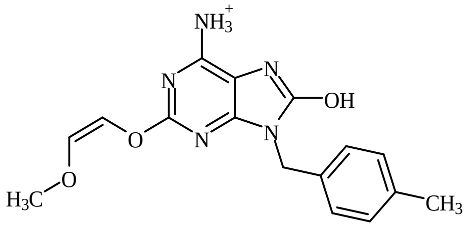
345.1678	$C_{16}H_{21}O_3N_6$	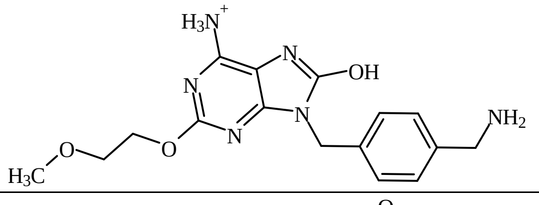
369.1311	$C_{17}H_{17}O_4N_6$	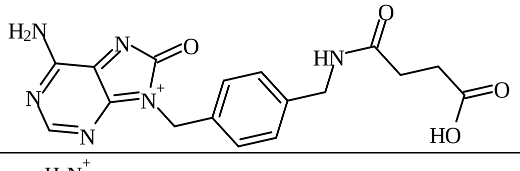
387.142	$C_{17}H_{19}O_5N_6$	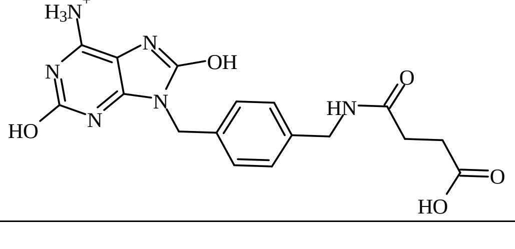

413.1572	$C_{19}H_{21}O_5N_6$	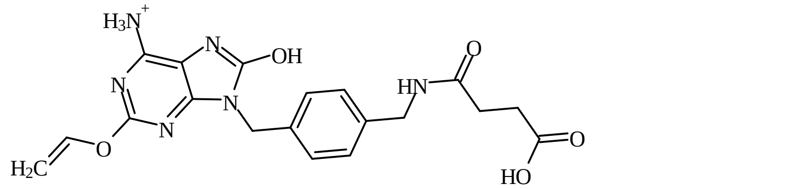

Table S7: Viability in undifferentiated (undiff.) and PMA-differentiated (diff.) THP-1 cells, measured with the Resazurin, LDH, and PI assays (values are expressed as mean ± standard deviation (n = 18), data are from three independent experiments).

	**Resazurin viability [%]**		**LDH viability [%]**		**PI viability [%]**	
	**undiff.**	**diff.**	**undiff.**	**diff.**	**undiff.**	**diff.**
1000 µM	91.4 ± 3.1	91.2 ± 4.7	102.1 ± 1.9	101.0 ± 2.2	101.8 ± 1.3	103.0 ± 15.8
500 µM	100.5 ± 3.7	97.0 ± 3.0	100.7 ± 1.3	100.8 ± 2.7	100.4 ± 1.6	102.0 ± 12.0
200 µM	104.8 ± 3.0	104.0 ± 4.0	99.9 ± 1.3	101.0 ± 1.9	99.8 ± 1.3	99.8 ± 6.6
100 µM	106.1 ± 4.7	105.2 ± 3.5	99.7 ± 1.0	101.2 ± 2.1	99.9 ± 1.1	99.6 ± 8.4
50 µM	106.3 ± 4.1	103.7 ± 4.9	100.1 ± 1.4	101.5 ± 1.7	100.5 ± 1.2	100.6 ± 4.9
20 µM	105.8 ± 3.0	105.1 ± 3.3	100.6 ± 0.7	101.6 ± 1.9	100.0 ± 1.4	102.3 ± 5.5
10 µM	105.0 ± 4.4	106.4 ± 4.2	100.7 ± 0.8	101.6 ± 2.0	99.7 ± 1.0	99.9 ± 4.0
untreated	100.0 ± 4.2	100.0 ± 4.2	100.0 ± 0.5	100.0 ± 0.2	100.0 ± 1.3	100.0 ± 2.3
20 % DMSO	0.0 ± 0.2	0.0 ± 0.3	n.a.	n.a.	0.0 ± 6.3	0.0 ± 5.8
1.2 % Triton-X-100	n.a.	n.a.	0.0 ± 10.9	0.0 ± 9.1	n.a.	n.a.

n.a. not applicable

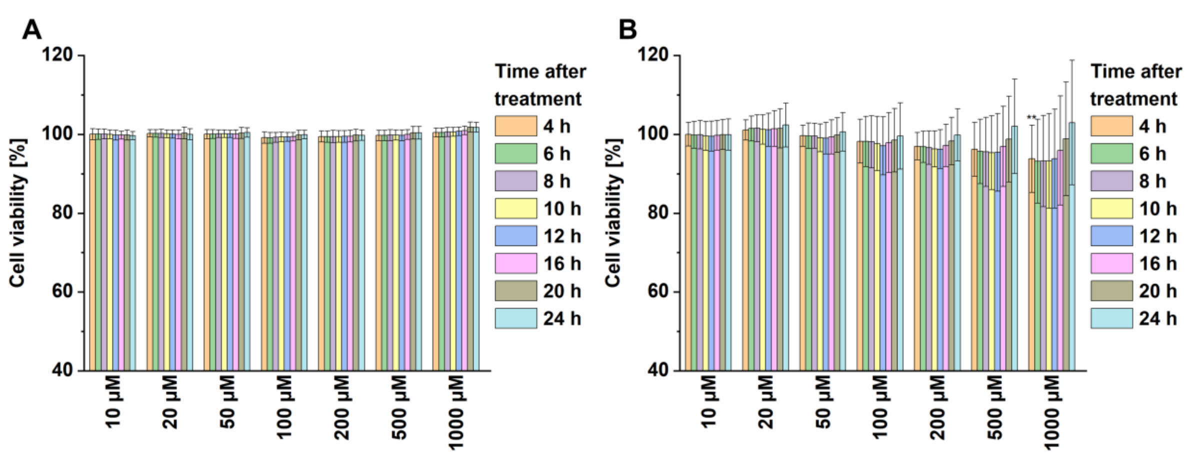

Fig. S7: Concentration-dependent cytotoxicity of SZU-106 on undifferentiated THP-1 cells (A) and PMA-differentiated THP-1 cells (B) at several time points, measured with PI-staining, mean of n = *18, error bars represent standard deviation, data from three independent experiments. The data were compared by the Kruskal-Wallis test to the untreated control samples*; *p<0.05; ***p*<0.01; ****p* <0.001).

Table S8: Cell viability in undifferentiated (monocyte-type) THP1-Dual-hTLR7 cells depending on the concentration of SZU-106, measured with lactate dehydrogenase (LDH)- and propidium iodide (PI)-assay. (values are expressed as mean ± standard deviation (n = 18), data were pooled from three independent experiments).

	**LDH viability [%]**	**PI viability [%]**
1000 µM	93.3 ± 3.9	82 ± 8.6
500 µM	94.6 ± 3.9	83.9 ± 8.4
200 µM	94.3 ± 3.9	85.3 ± 8.1
100 µM	95.6 ± 3.5	90.2 ± 7.0
50 µM	96.0 ± 3.6	89.8 ± 6.2
20 µM	98.4 ± 2.2	92.8 ± 5.1
10 µM	98.2 ± 2.3	93.5 ± 3.6
untreated	100.0 ± 0.6	100.0 ± 0.8
20 % DMSO	n.a.	0.0 ± 10.1
1.2 % Triton-X-100	0.0 ± 14.9	n.a.

n.a. not applicable

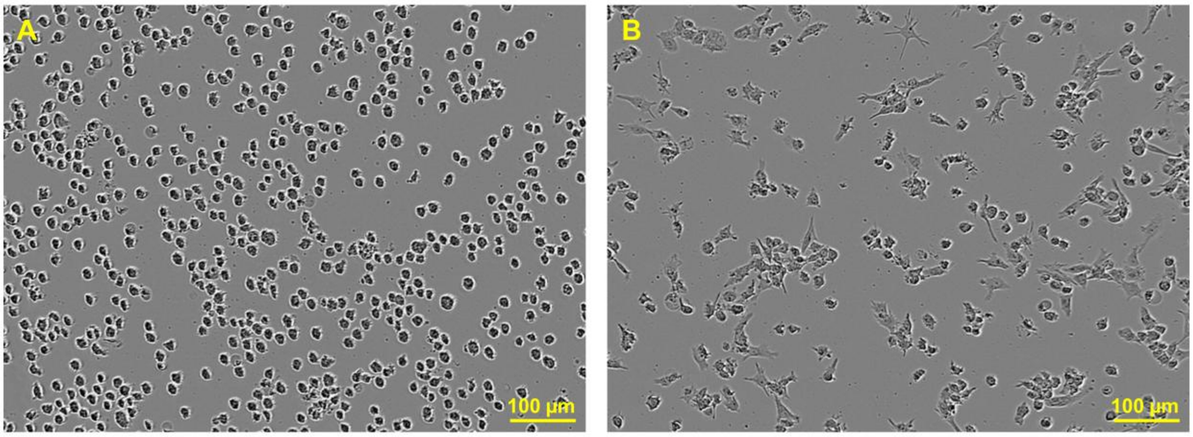

Fig. S8: Representative microscopic images of THP1-Dual-hTLR7 cells either untreated (A) or treated with 10 µM SZU-106 (B) after 24 h; scale bar: 100 µm

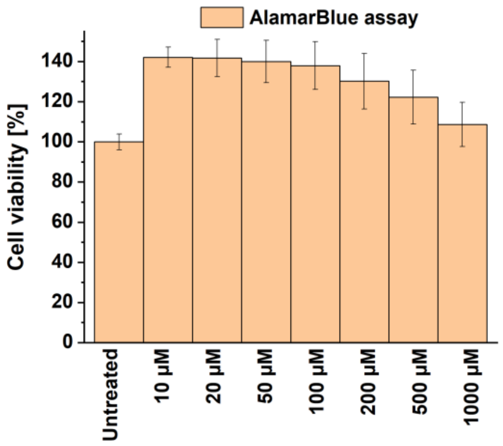

Fig. S9: Endpoint cell viability of THP1-Dual-hTLR7 with different concentrations of SZU-106 measured with resazurin assay, illustrating disturbance of the assay by increased reduction of resazurin compared to untreated cells. (mean of n = 18, error bars represent standard deviation; data from three independent experiments)

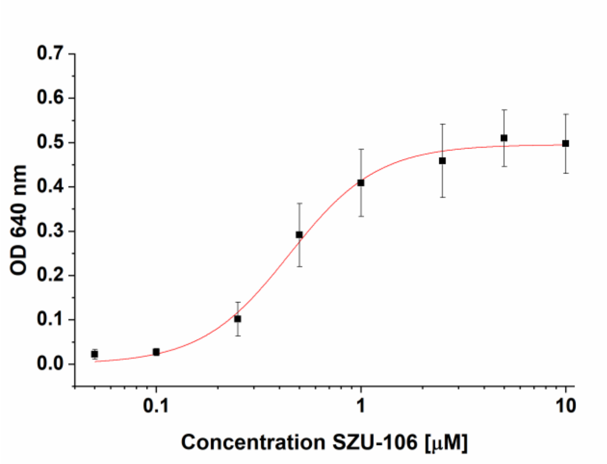

Fig. S10: NF-κB–dependent SEAP expression in THP1 Dual hTLR7 cells after 24 h of treatment with different concentrations of SZU-106. Red line represents a fit based on the equation: $y = \frac{y_{max}}{1+\left(\frac{EC_{50}}{x}\right)^n}$ *(mean of n = 18, error bars represent standard deviation).*

EC50 = 0.45 ± 0.02 µM
